# Biomechanical study of a biplanar double support screw (BDSF) technique based on Pauwels angle in femoral neck fractures: finite element analysis

**DOI:** 10.3389/fbioe.2024.1358181

**Published:** 2024-05-15

**Authors:** Zhongjian Tang, Yazhong Zhang, Shaolong Huang, Zhexi Zhu, Chengqiang Zhou, Ziqiang Zhu, Yunqing Wang, Bin Wang

**Affiliations:** ^1^ Department of Orthopaedics, The Second Affiliated Hospital of XuZhou Medical University, Xuzhou, China; ^2^ Graduate School of Xuzhou Medical University, Xuzhou, China

**Keywords:** femoral neck fracture, Pauwels angles, BDSF, hollow screw, finite element

## Abstract

**Objective:**

The objective of the present study is to conduct a comparative analysis of the biomechanical advantages and disadvantages associated with a biplanar double support screw (BDSF) internal fixation device.

**Methods:**

Two distinct femoral neck fracture models, one with a 30° angle and the other with a 70° angle, were created using a verified and effective finite element model. Accordingly, a total of eight groups of finite element models were utilized, each implanted with different configurations of fixation devices, including distal screw 150° BDSF, distal screw 165° BDSF, 3 CLS arranged in an inverted triangle configuration, and 4 CLS arranged in a “α” configuration. Subsequently, the displacement and distribution of Von Mises stress (VMS) in the femur and internal fixation device were assessed in each fracture group under an axial load of 2100 N.

**Results:**

At Pauwels 30° Angle, the femur with a 150°-BDSF orientation exhibited a maximum displacement of 3.17 mm, while the femur with a 165°-BDSF orientation displayed a maximum displacement of 3.13 mm. When compared with the femoral neck fracture model characterized by a Pauwels Angle of 70°, the shear force observed in the 70° model was significantly higher than that in the 30° model. Conversely, the stability of the 30° model was significantly superior to that of the 70° model. Furthermore, in the 70° model, the BDSF group exhibited a maximum femur displacement that was lower than both the 3CCS (3.46 mm) and 4CCS (3.43 mm) thresholds.

**Conclusion:**

The biomechanical properties of the BDSF internal fixation device are superior to the other two hollow screw internal fixation devices. Correspondingly, superior biomechanical outcomes can be achieved through the implementation of distal screw insertion at an angle of 165°. Thus, the BDSF internal fixation technique can be considered as a viable closed reduction internal fixation technique for managing femoral neck fractures at varying Pauwels angles.

## 1 Introduction

Femoral neck fractures (FNFs) constitute ∼3.6% of total body fractures and ∼50% of fractures occurring in the hip (Luo et al., 2023). Correspondingly, the treatment of femoral neck fractures in the young adult population remains a challenge in the field of orthopedic medicine. The proliferation of medical technology and the swift progress of minimally invasive surgery have led to a gradual expansion in the range of available internal fixation techniques (Al-Ani et al., 2015). Currently, the internal fixation techniques commonly employed for femoral neck fractures include the use of cannulated lag screw (CLS), dynamic hipscrew (DHS), dynamic condylar screw (DCS), and dynamic hipscrew (DHS). Among these, the DCS, femoral neck system (FNS), proximal femoral locking plates (PFLP), cutaneous compression plate (CCP), percutaneous compression plate (PCCP), medial support plate, and intramedullary fixation system fixation, along with other internal fixation devices, exhibit superior anti-rotation and anti-shear properties compared to hollow compression screws (Schwartsmann et al., 2017). However, their use may result in increased trauma and compromise the blood supply to the femoral head. Despite the high rate of femoral head necrosis and revision following internal fixation for femoral neck fractures in young adults, internal fixation is remains the favored treatment option (Samsami et al., 2015). Currently, the predominant treatment for femoral neck fractures in young adults is the closed reduction CLS internal fixation technique (Augat et al., 2019). Although accurately identifying and addressing CLS during surgical procedures poses challenges, the precise location and quality of fixation of CLS significantly impact clinical outcomes (Guo et al., 2020). Currently, there is considerable interest in investigating the variation in fixation efficacy among different CLS internal fixation techniques (Xiong et al., 2019). In recent years, the utilization of intraoperative navigation assistance technology in orthopedic surgery has become prevalent, driven by the demands of precision medicine. This technology has gained popularity due to its notable attributes of safety, accuracy, and efficiency in orthopedic surgical procedures (Thakkar et al., 2017). Furthermore, the advent of surgical robots has effectively addressed the challenge of precise positioning of CLS during surgical procedures. However, there remains a dearth of biomechanical studies investigating the fixation efficacy of various internal fixation techniques for CLS.

Currently, the CLS internal fixation technique employing 3 inverted triangles and the "α" configuration of 4 screws (3 screws fixed by the inverted triangle combined with 1 horizontal screw) is frequently used in the treatment of femoral neck fractures. However, several issues such as screw loosening, screw breakage, and weak anti-rotation force after surgery, affect the overall efficacy of this procedure [(Enocson and Lapidus, 2012; Samsami et al., 2015)]. In this context, Orlin Filipov introduced a novel technique for internal fixation known as the biplanar double support screw (BDSF). Herein, three CLS are strategically positioned on two vertically inclined planes, effectively reducing the stress load experienced by the internal fixation device at the site of femoral fracture (Filipov, 2019). In addition, it improved the stability of the femoral head under load, while also reducing the overall displacement of both the femur and the internal fixation device (Filipov, 2019). However, the implantation angle of the distal screw (from the top to the third CLS) in the BDSF internal fixation technique significantly affects its clinical efficacy (Tianye et al., 2019a; Lin et al., 2021). In this context, studies on the biomechanical effects resulting from changes in the implantation angle of the distal screw in the BDSF internal fixation device have been very limited. Currently, three different hollow internal fixation techniques have been reported in clinical studies, which have demonstrated satisfactory clinical efficacy. However, there is a dearth of corresponding biomechanical comparative analysis in the literature. Thus, the objective of the present study was to conduct a comparative analysis of the biomechanical advantages and disadvantages of three different CLS internal fixation techniques used in the treatment of femoral neck fractures. In addition, this study aimed to compare the biomechanical impact of altering the distal screw implantation angle on the treatment of femoral neck fracture using the BDSF internal fixation device.

## 2 Materials and methods

### 2.1 Data scanning and modeling

Healthy male volunteers (48 years old), having a minimum height of 173 cm, and weighing ∼75 kg were selected for the current study. The volunteers understood the underlying objective of the experimental study and proceeded to affix their signature on an informed consent form. The present research protocol was conducted in strict adherence to the ethical guidelines established by our institution. The Siemens 128-slice spiral CT scanner was provided by the Second Affiliated Hospital of Xuzhou Medical University. Thin-slice CT scanning of the volunteer’s pelvis and lower limbs was performed with a voltage of 120 kV, a current of 150 mA, and a scan layer thickness of 0.625 mm. The femoral model was reconstructed using threshold segmentation, region growth, and three-dimensional reconstruction features in Mimics 21.0 software (Materialise, Belgium). Simultaneously, the cancellous bone model was established in 3-matic12.0 software (Materialise, Belgium). The surface solid model of the femur was created using Ge-omagic12.0 software (Raindrop, United States).

### 2.2 Establishment of femoral neck fracture model

**FIGURE 1:**
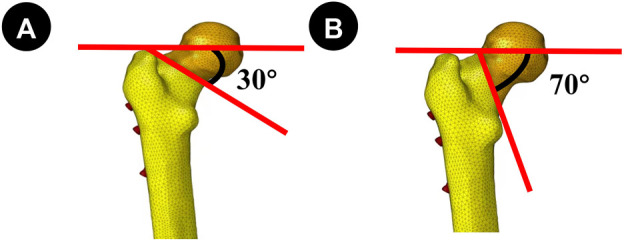
**(A, B)** Fracture lines were Pauwels 30° and 70°, respectively. Establishing the model for the internal fixation device.

**FIGURE 2:**
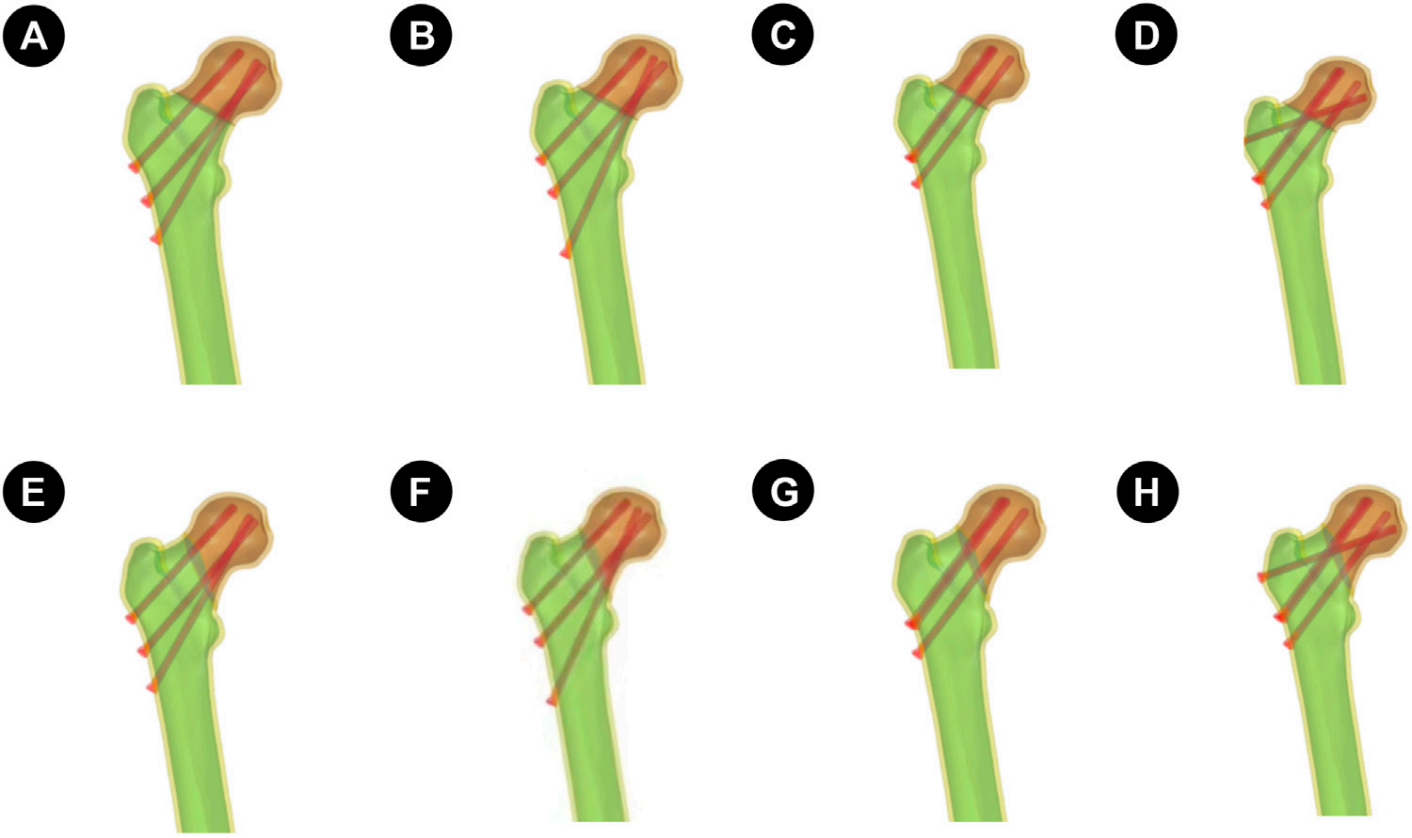
The four sets of finite element models are described as follows: **(A–D)** is the Pauwels 30° Angle femoral neck fracture model; E–H is the Pauwels70° Angle femoral neck fracture model. **(A and E)**: Implanted BDSF internal fixation device with distal screw at 150° Angle; **(B and F)**: The BDSF internal fixation device was implanted with the distal screw at a 165° Angle; **(C and G)**: 3 CLS internal fixation devices in inverted triangle configuration; **(D and H)**: 4 CLS internal fixation devices in “α” configuration.

The aforementioned models were imported into ProEngineer5.0 (PTC, the United States) software for smoothing, mesh generation, noise reduction, and surface adaptation to establish a three-dimensional solid model. Then, they were imported into SolidWorks 2017 software (Dassault Systèmes, France). Boolean operations were used to establish three-dimensional models of cortical and cancellous bones, creating femoral neck fracture models with Pauwels angles of 30° and 70° (Figure 1). Based on clinical procedures and engineering geometry data, four different fixation models were generated in Solidworks software: the distal screw 150° BDSF, distal screw 165° BDSF, inverted triangular, and “α”configuration CLS models (Figure 2).

Distal screw 150°BDSF Internal Fixation model (150°-BDSF):The three CLS were positioned along the lateral femoral wall, specifically below the greater trochanter of the femur. The proximal and middle two screws were positioned in a parallel manner to the femoral shaft at an angle of 135°, while the distal CLS screw was placed in close proximity to the femoral calaris at an angle of 150° relative to the femoral shaft. The distal end of the three CLS was observed to be approximately 5 mm distant from the articular surface (Figure 2).

Distal screw 165°BDSF Internal Fixation model (165°-BDSF):The three CLS were positioned along the lateral aspect of the femoral wall, specifically below the greater trochanter of the femur. The proximal and middle two screws were inserted in a parallel orientation to the femoral shaft at an angle of 135°, while the distal CLS screw was positioned near the femoral calaris at an angle of 165° relative to the femoral shaft. The distal end of the three CLS exhibited a spatial separation of approximately 5 mm from the adjacent articular surface (Figure 2).

Inverted Triangle Configuration 3-CLS Internal Fixation model (3CCS):The three CLS were arranged in a parallel configuration, forming an inverted triangle. Subsequently, the distal end of each CLS was positioned approximately 5 mm from the articular surface (Figure 2).

“α" Configuration 4 CLS Internal Fixation model (4CCS):The initial three CLS were arranged in a parallel configuration with an inverted orientation, while the final CLS was positioned slightly horizontally on the lateral femoral wall, specifically above the greater trochanter of the femur. The distal extremity of the four CLS was observed to be approximately 5 mm distant from the articular surface (Figure 2).

### 2.3 Material parameter settings and mesh generation

All experimental models were considered ideal continuous, uniform, isotropic linear elastic materials. According to previous studies (Taylor et al., 1996; Tianye et al., 2019b; Li et al., 2019), the material parameters for the finite element models in this study are presented in Table 1. The node and element counts for the four finite element models are shown in Table 2.

**TABLE 1:** Material parameters of the finite element model.

Materials	Young’s modulus (MPa)	Poisson’s ratio
Cortical bone	16,800.0	0.3
Cancellous bone	840.0	0.3
Head of femur	900.0	0.29
Collum femoris	620.0	0.29
Titanium alloy	110000.0	0.3

**TABLE 2:** The number of nodes and elements of the four finite element models.

Design	Unit	Node
30° Angle 150°-BDSF	5,71,492	8,33,854
30° Angle 165°-BDSF	5,64,758	8,25,790
30° Angle 3CCS	5,47,219	7,98,302
30° Angle 4CCS	6,22,944	9,13,219
70° Angle 150°-BDSF	5,64,264	8,22,172
70° Angle 165°-BDSF	5,75,359	8,41,852
70° Angle 3CCS	5,59,423	8,16,013
70° Angle 4CCS	6,31,475	9,26,498

### 2.4 Boundary conditions and loads

The force on the femur is complex. Under normal movement such as gait, the maximum load through the hip joint is 2.6–4.1 times the body weight. As step speed, step length, or body weight increase, the load on the hip joint also increases. The muscular forces acting on the femur are highly complex. Taylor et al. (Taylor et al., 1996) suggest that there are many uncertainties in modeling the femur with muscle loading, including the selection of muscle quantity, gravity, and the direction of muscle force loading, especially in dynamic simulations where accurate modeling is almost impossible. To simplify the analysis and highlight the fixation effects of the two groups of models, a vertical force of 2100N was applied to the femoral head to simulate human standing on two legs, one leg, and climbing stairs, according to literature references (Mei et al., 2014; Cui et al., 2020; Wang et al., 2020). Full constraints were applied to all nodes below the distal condyle of the femur.

### 2.5 Validity verification

To validate the effectiveness of the models, a complete femoral model was first established. Material properties were assigned following the methods described in the literature (Tsun et al., 2022; Papini et al., 2007). With the lower end of the model fully constrained and a vertical load of 2100 N applied to the femoral head, the model was analyzed using Ansys19.0 software (ANSYS, United States) and compared with the results reported in the literature (Tsun et al., 2022; Papini et al., 2007).

### 2.6 Evaluation metrics

Simulation calculations were performed in Ansys19.0 software, with the primary focus on comparing the maximum stress Von Mises distribution (VMS) and maximum deformation under a 2100N load condition between the two groups of models.

## 3 Results

### 3.1 Validity verification results

Under pure compression of 2100 N, the compression stiffness of the complete model was 0.798 kN/mm, which is very close to the reported compressive stiffness of [(0.76 ± 0.26) kN/mm] from the literature (Tsun et al., 2022; Papini et al., 2007). The stress distribution in the femur gradually increased from the distal end to the proximal end, with the highest stress peak located at the femoral neck, consistent with the findings of Yan Shigui et al. (Zhan et al., 2020). Considering the individual differences among models, the models established in this experiment are considered valid.

### 3.2 Von mises stress (VMS) distribution and results of four CLS internal fixation devices and femur

**FIGURE 3:**
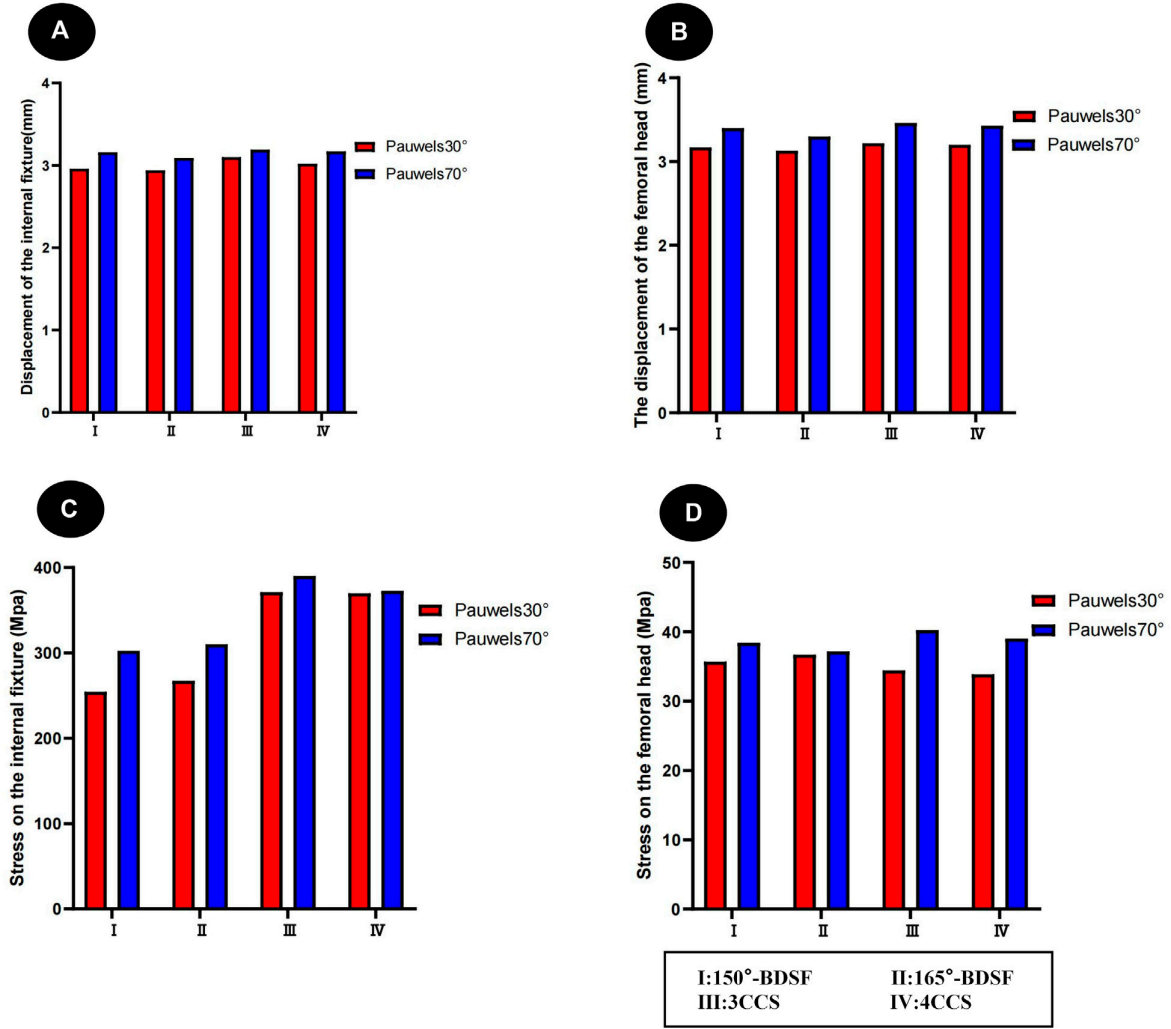
Distribution trends of displacement and stress results for two fracture types fixed by four internal fixation devices. **(A)** Results of the femur displacement distribution of four internal fixation devices; **(B)** Results of the femur displacement distribution of four internal fixation devices; **(C)** Results of the femoral stress distribution in four internal fixation devices; **(D)** Results of the femoral stress distribution in four internal fixation devices.

**FIGURE 4:**
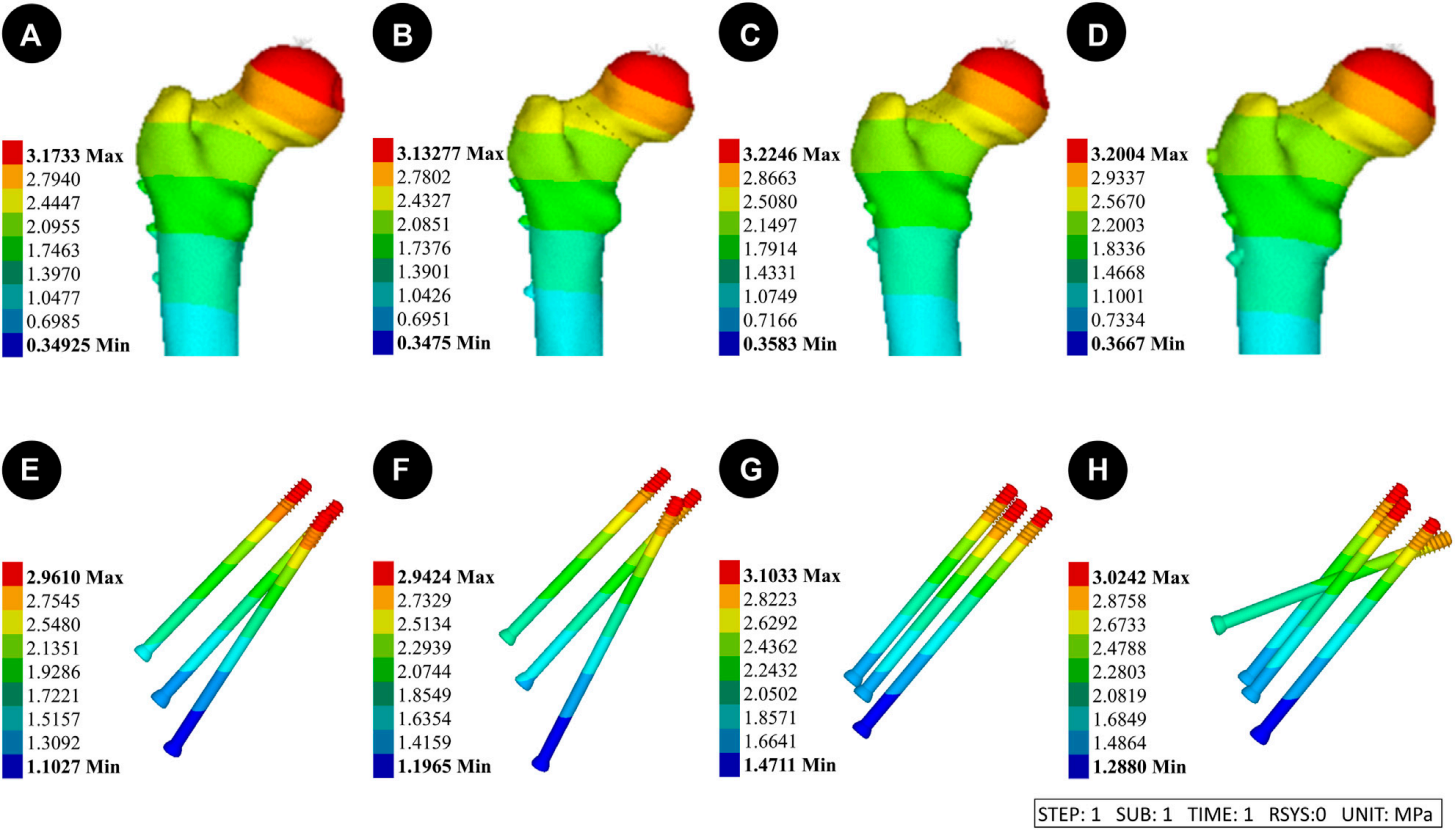
Results of the femur displacement distribution of four internal fixation devices at Pauwels 30° Angle. **(A and E)**: Implanted BDSF internal fixation device with distal screw at 150° Angle; **(B and F)**: The BDSF internal fixation device was implanted with the distal screw at a 165° Angle; **(C and G)**: 3 CLS internal fixation devices in inverted triangle configuration; **(D and H)**: 4 CLS internal fixation devices in "α" configuration.

Figure 3 Pauwels 30° Angle FNFs: The maximum value of VMS in the femur was recorded as 35.7 MPa at an angle of 150°-BDSF, 36.69 MPa at an angle of 165°-BDSF, 34.14 MPa under 3CCS loading conditions, and 33.86 MPa under 4CCS loading conditions. The maximum values of the peak VMS for the internal fixers, viz., the 150°-BDSF, 165°-BDSF, 3CCS, and 4CCS, were recorded as 254.43 MPa, 267.29 MPa, 371.24 MPa, and 369.99 MPa, respectively. Based on the displacement profile analysis of the Pauwels fracture at a temperature of 30° FNFs, it was observed that the maximum VMS in the femur was located in the cortical region below the fracture. Additionally, the VMS in the internal fixation device was found to be concentrated on the surface of the screw in close proximity to the fracture line (Figure 4).

**FIGURE 5:**
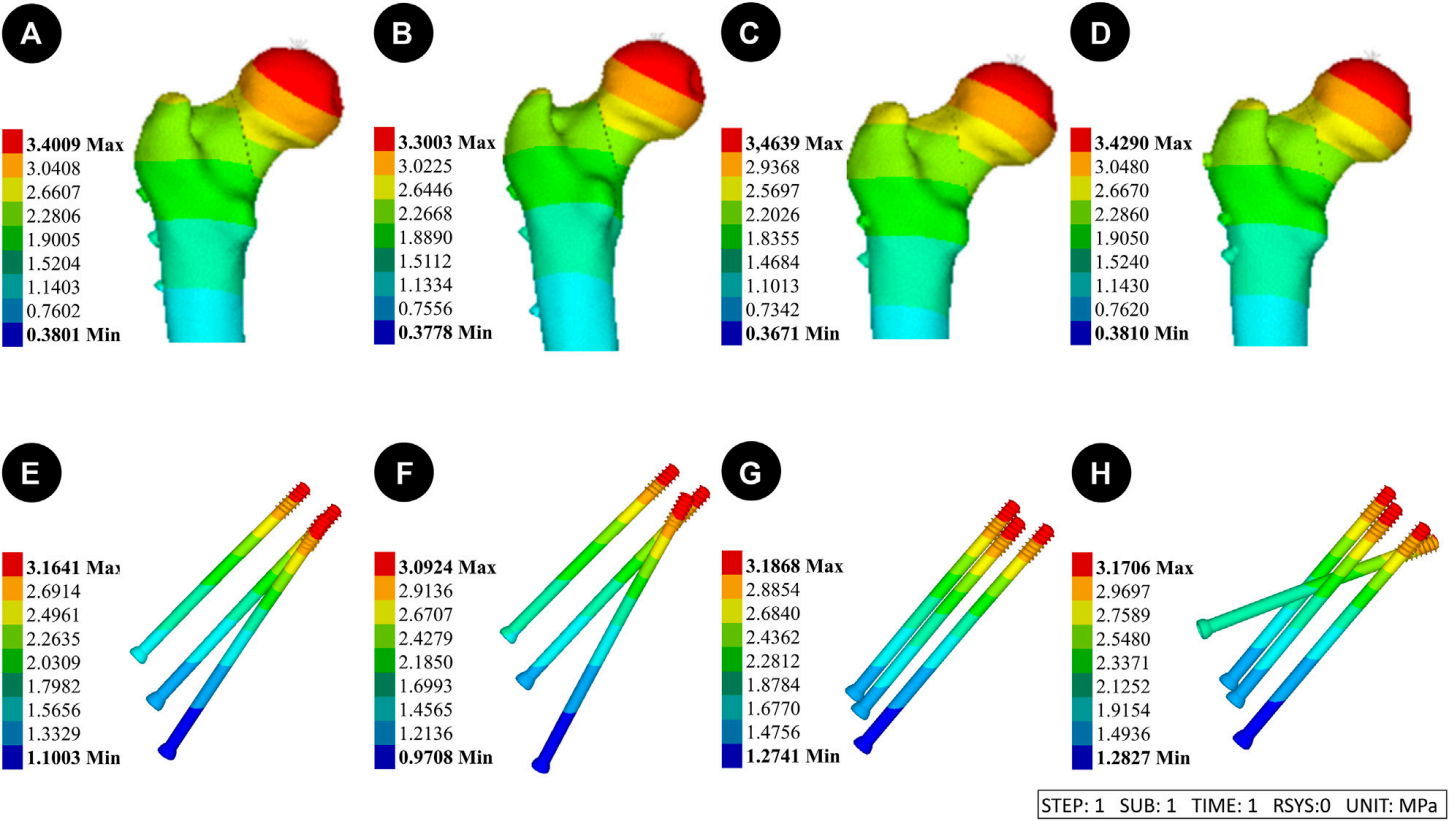
Results of the femur displacement distribution of four internal fixation devices at Pauwels 70° Angle. **(A and E)** Implanted BDSF internal fixation device with distal screw at 150° Angle; **(B and F)** The BDSF internal fixation device was implanted with the distal screw at a 165° Angle; **(C and G)** 3 CLS internal fixation devices in inverted triangle configuration; **(D and H)** 4 CLS internal fixation devices in “α” configuration.

Pauwels 70° Angle FNFs: The peak VMS for the 150°-BDSF femur was measured to be 38.42 MPa, while for the 165°-BDSF femur it was found to be 37.16 MPa. The 3CCS femur exhibited a peak VMS of 40.24 MPa, whereas the 4CCS femur had a peak VMS of 39.03 MPa. In addition, the peak VMS values for the internal fixtures, *viz.*, the 150°-BDSF, 165°-BDSF, 3CCS, and 4CCS, were recorded as 302.59 MPa, 310.24 MPa, 390.28 MPa, and 372.61 MPa, respectively. Based on the displacement profile analysis of the Pauwels fracture at a temperature of 70° FNFs, it was observed that the VMS in the femur were primarily localized in the cortical region below the fracture. Additionally, the VMS were found to be concentrated on the surface of the screw in close proximity to the fracture line (Figure 5).

### 3.3 Displacement results and distribution of four CLS internal fixation devices and femur

**FIGURE 6:**
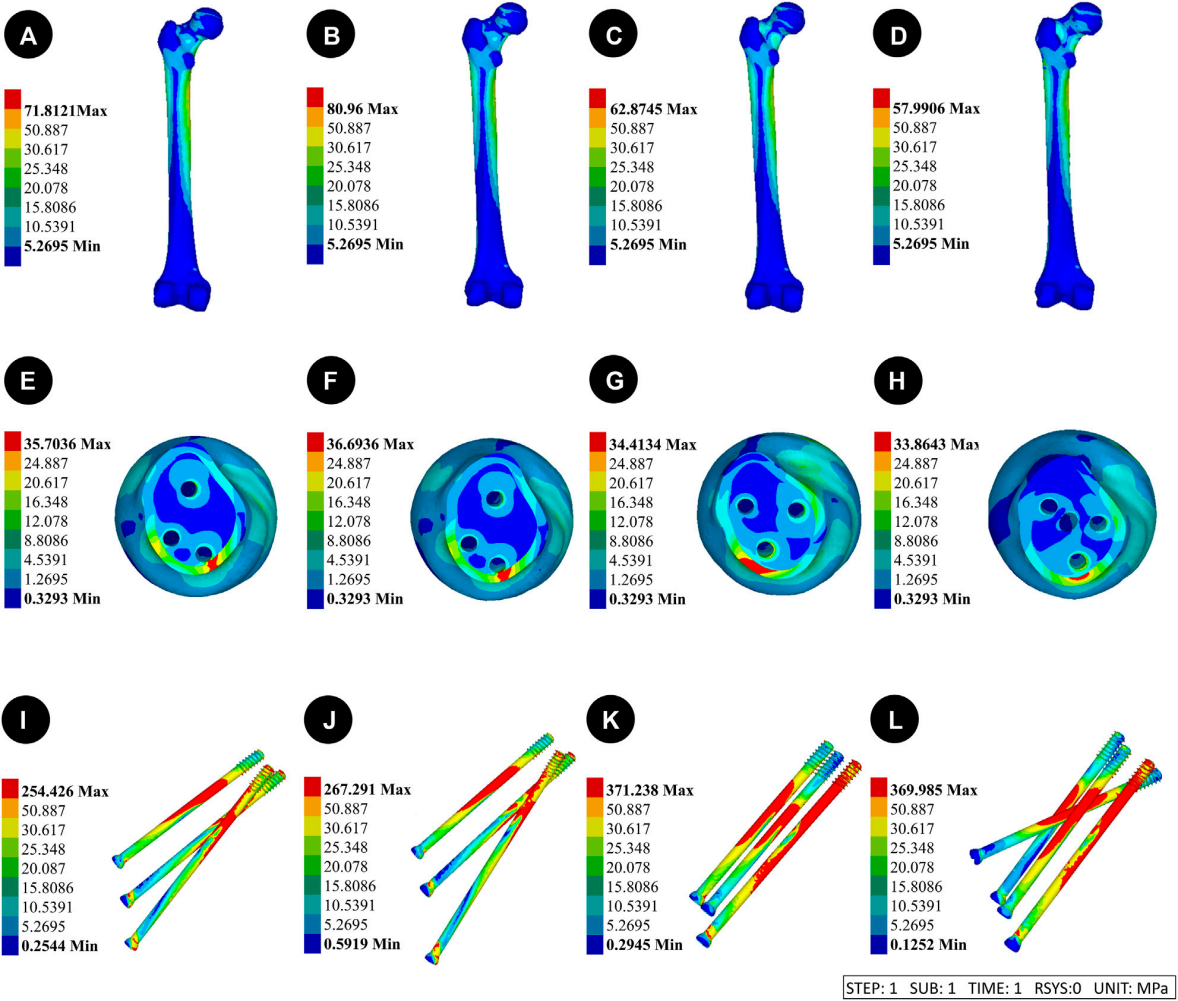
Results of the femoral stress distribution in four internal fixation devices at Pauwels 30° Angle. **(A, E and I)** Implanted BDSF internal fixation device with distal screw at 150° Angle; **(B, F and J)** The BDSF internal fixation device was implanted with the distal screw at a 165° Angle; **(C, G and K)** 3 CLS internal fixation devices in inverted triangle configuration; **(D, H, L)** 4 CLS internal fixation devices in “α” configuration.

(Figure 3) Pauwels 30° Angle FNFs: The femur with a 150°-BDSF exhibited a maximum displacement of 3.17 mm, while the femur with a 165°-BDSF had a maximum displacement of 3.13 mm. Similarly, the 3CCS femur and the 4CCS femur displayed maximum displacements of 3.22 mm and 3.20 mm, respectively. In addition, the internal fixtures, viz., the 150°-BDSF, 165°-BDSF, 3CCS, and 4CCS, exhibited maximum displacements of 2.96 mm, 2.94 mm, 3.12 mm, and 3.02 mm, respectively. Based on the displacement profile of the Pauwels fracture at 30°FNFs, it was observed that the highest displacement was observed above the femoral head. Additionally, the internal fixation device exhibited its maximum displacement at the uppermost part of the screw (Figure 6).

**FIGURE 7:**
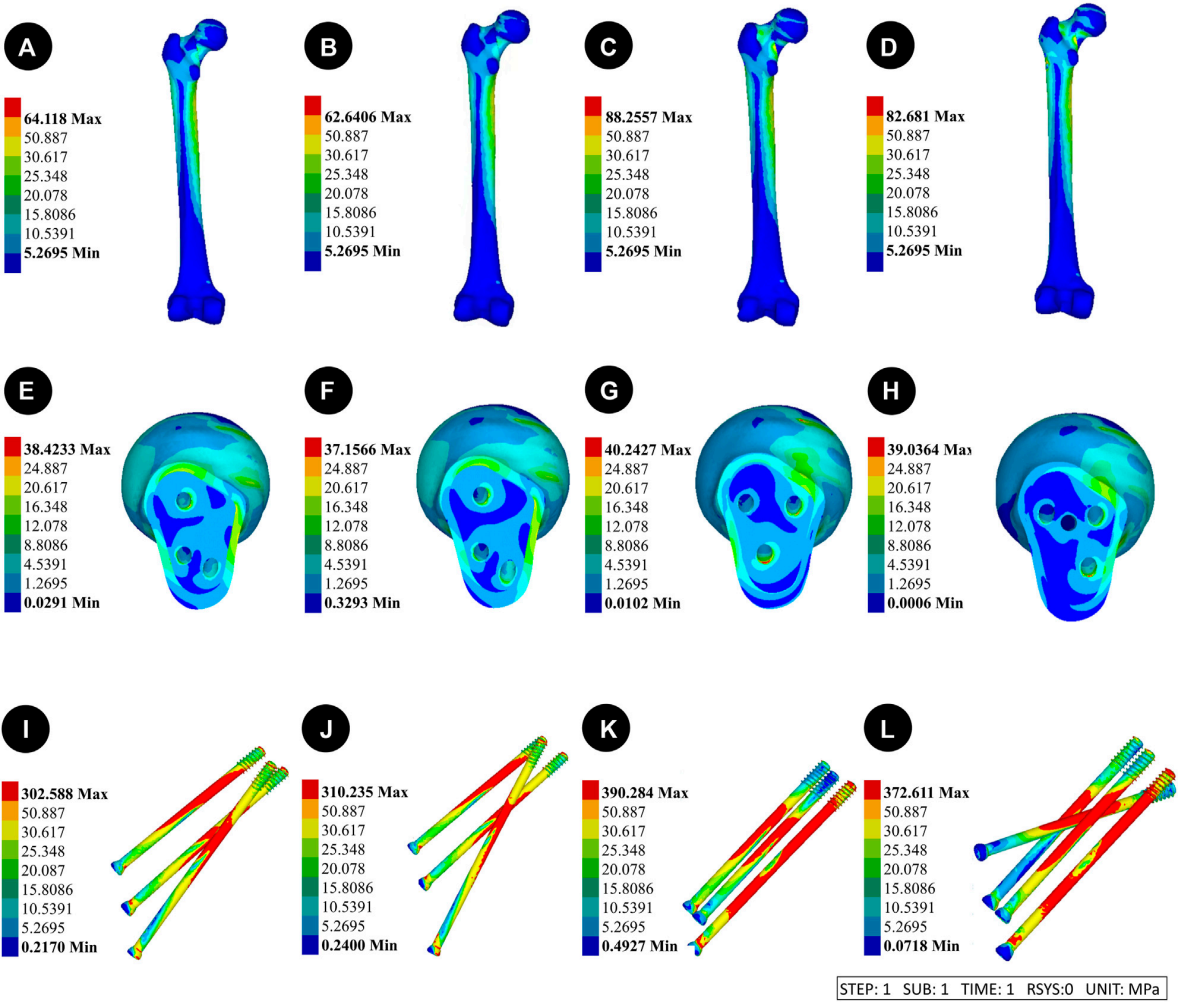
Results of the femoral stress distribution in four internal fixation devices at Pauwels 70° Angle. **(A, E and I)** Implanted BDSF internal fixation device with distal screw at 150° Angle; **(B, F and J)** The BDSF internal fixation device was implanted with the distal screw at a 165° Angle; **(C, G and K)** 3 CLS internal fixation devices in inverted triangle configuration; **(D, H, L)** 4 CLS internal fixation devices in “α” configuration.

Pauwels 70° Angle FNFs: The femur with a 150°-BDSF exhibited a maximum displacement of 3.40 mm, while the femur with a 165°-BDSF showed a maximum displacement of 3.30 mm. Similarly, the 3CCS femur had a maximum displacement of 3.46 mm, while the 4CCS femur had a maximum displacement of 3.43 mm. Additionally, the 150°-BDSF internal fixation exhibited a maximum displacement of 3.16 mm, while the 165°-BDSF internal fixation demonstrated a maximum displacement of 3.09 mm. Similarly, the 3CCS internal fixation displayed a maximum displacement of 3.19 mm, and the 4CCS internal fixation showcased a maximum displacement of 3.17 mm. Based on the displacement profile analysis of the Pauwels fracture at 70° FNFs, it was observed that the highest displacement was observed above the femoral head. Additionally, the internal fixation device exhibited its maximum displacement at the uppermost part of the screw (Figure 7).

## 4 Discussion

In recent years, the proliferation of computer technology has led to the widespread adoption of the finite element method as the predominant stress analysis technique in the field of biomechanics research. The application of mechanical simulation through finite element modeling enables the assessment of mechanical characteristics pertaining to fracture internal fixation devices. This approach offers a reliable basis for the discernment and selection of appropriate internal fixation techniques for femoral neck fractures. In young adults, femoral neck fractures typically exhibit a high degree of instability when subjected to vertical shear stress of high energy [(Flury et al., 2020; Cui et al., 2020)]. Moreover, the level of fracture perpendicularity directly correlates with the challenge of achieving adequate stability to withstand the vertical shear force exerted around the hip. The results of this study further confirm the existence of a favorable association between the Pauwels Angle and both the vertical shear force and the maximum displacement of the fracture surface in the two models of femoral neck fractures. Moreover, the VMS in the femur tend to concentrate in the cortical region below the fracture, while the VMS in the internal fixation device also exhibit a similar concentration. These observations suggest an increased likelihood of femoral refracture and internal fixation device fracture.

Herein, three CLS internal fixation devices were utilized in an inverted triangle configuration. The utilization of the three CLS aligned in parallel to the femoral calaris represents a prevalent technique for internally fixing femoral neck fractures in the young adult population. The percutaneous implantation of three CLS offers several advantages, including reduced trauma and shorter operation time. However, the use of an inverted triangle configuration CLS for the treatment of femoral neck fractures has been reported to result in postoperative complications in 20%–48% of cases [ (Filipov, 2011; Wang et al., 2020)]. The fixation of the CLS in alignment with the femoral calaris is suitable for Pauwels type I femoral neck fractures characterized by a small angle between the fracture line and the horizontal line. However, this fixation technique is not suitable for Pauwels type II and type III femoral neck fractures, which exhibit a large angle between the fracture line and the horizontal line. In such cases, using this technique is likely to result in postoperative fracture nonunion and failure of the internal fixation device [(Liporace et al., 2008; Marsh et al., 2007; Mei et al., 2014; Tsun et al., 2022)].

Several studies have indicated that the “α” configuration of four CLS internal fixation technology offers more advantages compared to three CLS internal fixation devices in an inverted triangle configuration [(Kauffman et al., 1999; Papini et al., 2007)]. This specific configuration is ideally suited for the compression fixation of fracture ends, as it demonstrates exceptional resistance to vertical shear forces. A comprehensive study conducted by Kauffman revealed that when dealing with comminuted femoral posterior neck fractures, the utilization of a 4-CLS fixation device significantly enhances stability when compared to the traditional 3-CLS internal fixation device (Zhan et al., 2020; Biraris et al., 2014). Additionally, the clinical effectiveness of the 4-CLS fixation device was superior to that of the inverted triangle configuration CLS. As observed, the findings of this study align with the conclusions drawn from prior research. Moreover, in Pauwels 30° Angle fracture model, no notable biomechanical distinctions between 3 CLS internal fixation devices arranged in an inverted triangle configuration and the use of 4 screw internal fixation devices arranged in a "α" configuration for the purpose of stabilizing femoral neck fractures were observed. However, when the fracture model Angle was adjusted to Pauwels 70°, it was observed that the vertical shear force of the fracture increased, leading to a decrease in stability. Furthermore, it was found that the “α” configuration 4-screw internal fixation device exhibited superior maximum displacement and VMS compared to the inverted triangle 3-CLS internal fixation device. Herein, it was observed that the stability of 4 CLS surpassed that of 3 CLS techniques. This superiority can be attributed to the fourth transverse CLS, which created a larger Angle with the fracture surface. This angle facilitated the application of pressure to the fracture end and contributed to the alleviation of a portion of the vertical shear force. However, in the case of Pauwels 30° Angle fracture, the orientation of the fracture line is more horizontally aligned compared to the 70° Angle fracture. As a result, the Angle between the fourth transverse CLS and the fracture line was smaller, leading to a decrease in the compression fixation effect and anti-shear ability. Consequently, the biomechanical advantages and disadvantages of the two internal fixation devices are not clearly discernible.

The BDSF technique is a newly emerged CLS internal fixation technique. Herein, 3 CLS are positioned on two vertically inclined planes. Specifically, the distal screw is placed on the anticlinal plane, while the middle screw and the proximal screw are situated on the ventral oblique plane. The distal screw serves as the primary supportive screw, anchoring the distal cortex of the femoral neck and the lateral cortex of the femoral shaft [(Al-Ani et al., 2015; Flury et al., 2020)]. Furthermore, it offers additional support to the posterior cortex of the femoral neck [(Al-Ani et al., 2015; Flury et al., 2020)]. Earlier studies have indicated that the use of BDSF, in comparison to conventional multi-parallel CLS internal fixation methods, can offer enhanced support for the posterior and lower cortex of the femoral neck [(Zhang et al., 2022; Filipov, 2011)]. This, in turn, leads to improved overall stability and resistance against shearing forces of the internal fixation device [(Zhang et al., 2022; Filipov, 2011)]. The findings of this study deviate slightly from those of previous studies. Herein, the Pauwels 30° Angle fracture model was utilized to examine the femoral neck fracture treated with the "α" configuration using a 4-screw internal fixation device. It was observed that this treatment approach resulted in reduced stress on the femoral head compared to the BDSF group. However, it was also found that the maximum displacement of the femur and the stress on the internal fixation device were significantly higher in the “α” configuration group compared to the BDSF group. The transition to Pauwels 70° Angle fracture type resulted in a notable increase in the overall VMS of the BDSF internal fixation device. However, even with this increase, the VMS remained considerably lower than that of the “α” configuration 4-screw internal fixation device. This reduction in VMS significantly mitigated the risk of CLS fracture. The BDSF group exhibited significantly smaller maximum displacement and VMS of the femur compared to the "α" configuration 4-screw internal fixation device. This resulted in improved overall stability of the femur and reduced VMS of the femoral head, thereby creating a favorable mechanical environment for the healing of femoral neck fractures. In the present study, it was observed that when the “α” configuration of four screws was chosen for the purpose of fixation, 3 CLS exhibited an approximate angle of 130°, while the fourth CLS was inserted horizontally at a distance of less than 7 mm. This particular arrangement has the potential to result in excessive stress concentration and subsequent failure of the internal fixation. In comparison to the “α” configuration 4-screw internal fixation device, the BDSF internal fixation device features a CLS head that is positioned approximately 20 mm away from the femoral head. This strategic placement serves to effectively mitigate stress concentration and minimize the likelihood of subtrochanteric fracture. Furthermore, the internal fixation device known as the BDSF is designed in the “F” configuration, which offers a dual plane that effectively stabilizes the fractured end by facilitating cross support between the CLS. It also efficiently transmits the vertical stress resulting from body weight to the lateral femoral cortex, thereby enhancing the femoral neck’s resistance to axial compression. Moreover, the distal screw has the potential to offer both transverse and medial support, thereby potentially decreasing the strain experienced by the lateral bone cortex.

Several studies have indicated that the optimal placement of the distal screw plays a crucial role in achieving successful internal fixation in BDSF. Thus, satisfactory clinical outcomes can be achieved by placing distal screws along the lower and posterior cortex of the femoral neck at an obtuse angle with the shaft axis [(Tianye et al., 2019a; Liporace et al., 2008)]. According to Kloen et al., it is also suggested that the utilization of low angle distal screw fixation can effectively maintain the sliding pressure mechanism at the fracture site, minimize shear stress at the fracture site, and enhance the stability and shear resistance of the internal fixation device [(Fisher et al., 2023; Marsh et al., 2007)]. The findings of this study indicate that the stability and shear resistance of the two groups of BDSF internal fixation devices were found to be superior when compared to the inverted triangle 3 CLS internal fixation devices and the “α” configuration 4 screws internal fixation devices. In our analysis, it was determined that within the framework of the Pauwels 30° fracture model, the implementation of distal screws at both 150° and 165° angles exhibits the potential to enhance fixation strength and augment stability against axial, rotational, and bending stresses. The biomechanical distinction between the two implantation angles becomes evident as the fracture angle approaches Pauwels 70°. Subsequently, the angle formed between the distal screw’s implantation angle of 165° and the shaft axis increases, resulting in enhanced resistance of the distal screw against femoral neck inversion collapse. This increased resistance can effectively prevent complications associated with the CLS incision technique, while also providing stronger support to the posterior cortical bone. The transfer of axial load to the bone cortex enhances the load-bearing capacity of the screw and contributes to the preservation of both the femur’s overall stability and the internal fixation device.

The results of this study comprehensively illustrate the advantages and disadvantages associated with the utilization of three distinct internal fixation devices in CLS procedures. Furthermore, the investigation delves into the mechanical disparities among BDSF groups, particularly those exhibiting varying angles of distal screw implantation. These findings offer valuable insights for surgeons in terms of selecting appropriate surgical methods and determining optimal screw implantation trajectories for orthopedic robots. However, several limitations are present in this study: 1) The implantation angles of the distal screw were limited to 150° and 165°, and a definitive range of implantation angles could not be established. The applicability of fracture types and internal fixation devices was restricted to a particular study population, and the findings do not provide evidence for the use of other fracture types and internal fixation devices. 2) The material parameters of the model were defined as isotropic elastic materials, in contrast to the anisotropic material properties exhibited by real human bones. The objective of this study was to construct a model that represents the general pattern, thus making the chosen methodology justifiable. 3) The load setting solely replicates the load-bearing capability of an individual leg during the typical ambulation of adults, without taking into account the distinct function of each muscle group.

## 5 Conclusion

In FNFs with Pauwels 30° Angle or Pauwels 70° Angle, the BDSF internal fixation device demonstrated superior biomechanical performance compared to the inverted triangle configuration with 3 screws and the “α” configuration with 4 CLS internal fixation devices. Furthermore, distal screw insertion at a 165° Angle was observed to yield superior biomechanical outcomes. Thus, the BDSF internal fixation technique is a dependable closed reduction internal fixation method for treating fractures at various Pauwels angles.

## Data Availability

The original contributions presented in the study are included in the article/Supplementary Material, further inquiries can be directed to the corresponding author.
